# The Identification of Protein Kinase C Iota as a Regulator of the Mammalian Heat Shock Response Using Functional Genomic Screens

**DOI:** 10.1371/journal.pone.0011850

**Published:** 2010-07-29

**Authors:** Frank Boellmann, Russell S. Thomas

**Affiliations:** Center for Genomic Biology and Bioinformatics, The Hamner Institutes for Health Sciences, Research Triangle Park, North Carolina, United States of America; Duke-NUS Graduate Medical School, Singapore

## Abstract

**Background:**

The heat shock response is widely used as a surrogate of the general protein quality control system within the cell. This system plays a significant role in aging and many protein folding diseases as well as the responses to other physical and chemical stressors.

**Methods/Principal Findings:**

In this study, a broad-based functional genomics approach was taken to identify potential regulators of the mammalian heat shock response. In the primary screen, a total of 13724 full-length genes in mammalian expression vectors were individually co-transfected into human embryonic kidney cells together with a human HSP70B promoter driving firefly luciferase. A subset of the full-length genes that showed significant activation in the primary screen were then evaluated for their ability to hyper-activate the HSP70B under heat shock conditions. Based on the results from the secondary assay and gene expression microarray analyses, eight genes were chosen for validation using siRNA knockdown. Of the eight genes, only PRKCI showed a statistically significant reduction in the heat shock response in two independent siRNA duplexes compared to scrambled controls. Knockdown of the PRKCI mRNA was confirmed using quantitative RT-PCR. Additional studies did not show a direct physical interaction between PRKCI and HSF1.

**Conclusions/Significance:**

The results suggest that PRKCI is an indirect co-regulator of HSF1 activity and the heat shock response. Given the underlying role of HSF1 in many human diseases and the response to environmental stressors, PRKCI represents a potentially new candidate for gene-environment interactions and therapeutic intervention.

## Introduction

The regulation of the eukaryotic heat shock response has held considerable interest within the scientific community ever since the discovery of newly formed puffs in temperature-shocked Drosophila polytene chromosomes [1]. The speed, magnitude, and proportionality of the response has greatly aided in the identification of its basic regulatory scheme [2], [3]. The main regulatory proteins of the mammalian heat shock response are a group of molecular chaperones called heat shock proteins (HSPs) and the stress-activated transcription factor HSF1 [4], [5]. Heat shock proteins that function as molecular chaperones recognize misfolded proteins by binding hydrophobic peptide domains that are normally buried inside of properly folded proteins and assist in refolding or degradation [6], [7], [8]. Under steady-state conditions, HSF1 is sequestered in the cytosol of unstressed cells as part of a HSP90-containing multi-chaperone complex that keeps the transcription factor in a monomeric, inactive state [9]. Increasing amounts of alternative chaperone substrates lead to the release of HSF1 from the chaperone complex and its subsequent accumulation as a homo-trimeric protein in the nucleus of stressed cells [10], [11]. Trimerization is required for HSF1 to attain high binding affinity to heat shock elements (HSE), the specific binding sites in the promoters of heat shock genes [12]. The increase in transcriptional competence of HSF1 is accompanied by stress-induced phosphorylation at multiple serine residues [13]. Despite the identification of several protein kinase inhibitors that reduce HSF1 activity, no specific protein kinase and its corresponding HSF1 residue has been identified that is required for the full activation of the transcription factor [3], [14], [15].

The heat shock response can be rapidly activated following proteotoxic stress by responding to the rate of change in the abundance of denatured proteins. When this rate of change is too slow for the heat shock response to recognize and repair the damage, system failure in the form of protein folding diseases and aging is possible [16]. In addition, the negative feedback regulation of the stress response can have a significant impact on the dose response characteristics of the system following exposure to chemical and physical stressors [17]. As a result, identifying and characterizing all the genes that play a role in the heat shock signaling pathway is of interest in both toxicology and pharmacology [18], [19].

Despite the completion of the human genome sequence, the functional role of many genes and their organization into signaling pathways remains relatively unknown. A number of research groups have applied large-scale, reverse genomic screens to systematically identify genes that play a functional role in specific disease pathways and assign putative molecular roles to previously uncharacterized genes [20], [21], [22], [23]. In these screens, cell-based assays were constructed with various cellular endpoints [21] or reporter genes that indicate activation of a specific pathway [20], [22], [23], [24]. Expression plasmids containing full-length genes were then introduced into the cells to manipulate expression and the effects on the endpoint were evaluated. Since a single cell line may not express all potential regulators of the heat shock response, an overexpression strategy was employed using a series of cell-based screens. A subset of the regulators was then subjected to functional validation using RNAi. Based on these studies, protein kinase C iota (PRKCI) was identified as a regulator of the heat shock signaling pathway.

## Results

### Primary Screen for Activation of the Heat Shock Response Under Non-Stressed Conditions

**Figure 1:**
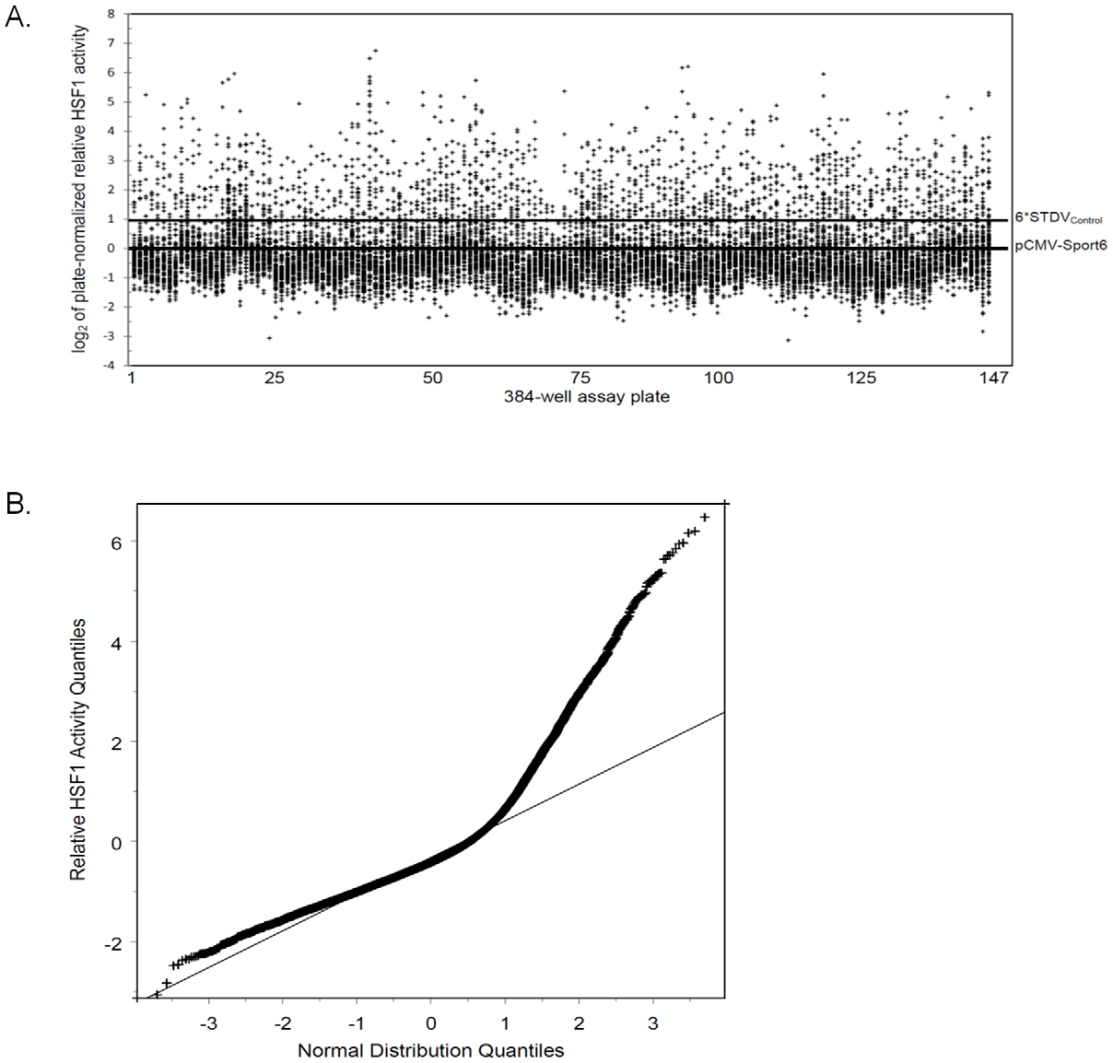
Summary results for the primary gain-of-function screen for activators of HSF1. A total of 13724 full-length genes were co-transfected with a human HSP70B promoter driving firefly luciferase and a constitutively expressed *Renilla* luciferase control. (A) Relative activity of the 13724 full-length genes normalized to the negative plasmid control on each plate (pCMV-Sport6 Empty). Each data point represents the average of four independent replicates. The line labeled 6*STDV_Control_ represents a cut-off of 6 standard deviations above the mean of the negative control. (B) The quantile distribution of the 13724 data points from the primary screen compared to a normal distribution (straight line).

An arrayed expression library containing 8674 mouse and 5050 human full-length genes in the mammalian expression vector pCMV-Sport6 was screened for inducers of HSF1 activity. Based on the Homologene database, 2501 orthologs are shared between the human and mouse libraries. Individual genes were transiently transfected together with the HSP70B luciferase reporter and the internal *Renilla* luciferase control plasmid into human embryonic kidney HEK-293T cells. The HSP70B promoter contains 3 full heat shock elements (NGAANNTTCN)[25]. Co-transfection of the constitutively expressed *Renilla* luciferase was performed to control for differences in transfection efficiency, cell viability and other non-specific effects. Variations in plate handling were corrected by normalization against the average of the negative control wells on each plate transfected with the empty pCMV-Sport6 plasmid (Fig. 1A). A total of 1001 full-length genes passed the criteria for selection in the primary screen. This number was reduced to 940 full-length genes after the elimination of multiple tubulin isoforms and species redundant low scoring proteins. A graph of the quantile distribution of the relative activity of all genes in the library was compared to a normal distribution (Fig. 1B). The deviation of the screening results above the straight line in the quantile-quantile plot indicates that a large portion of the library activates the HSP70B reporter at a higher frequency than one would expected from a random distribution. This suggests that the conditions used in the primary screen resulted in a large number of hits, but may have also resulted in an increased number of false positives. To reduce potential false positives, a secondary screen was performed to identify genes that hyper-activate the heat shock response under stress conditions.

### Secondary Screen for Hyper-Activation of the Heat Shock Response Under Stressed Conditions

**Figure 2:**
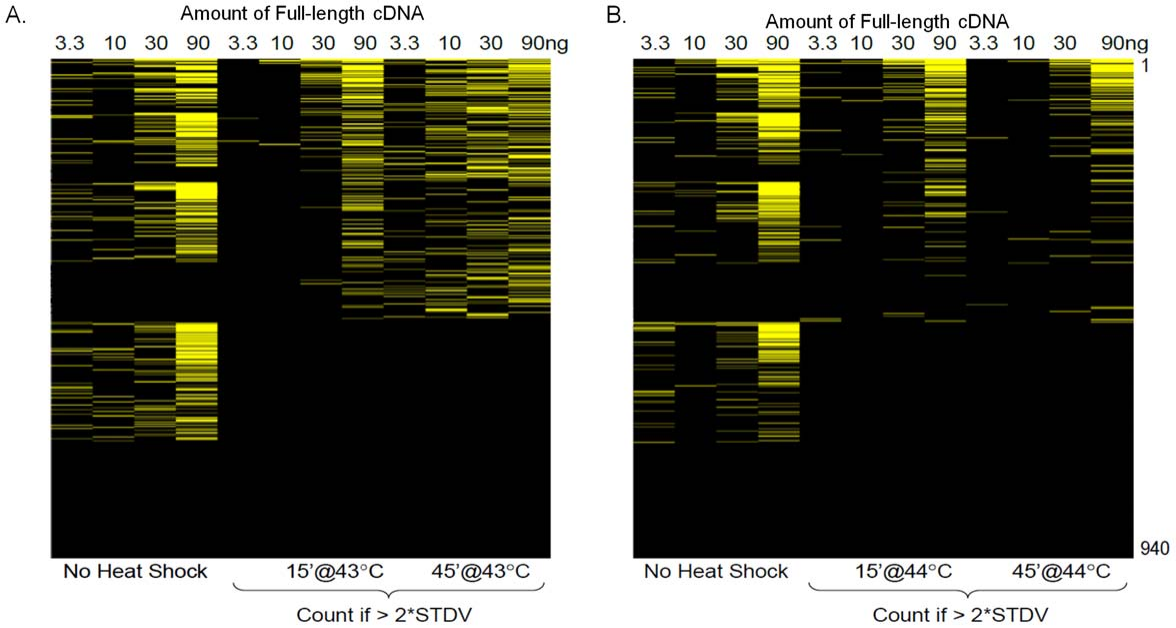
A heat map showing the results from the secondary screen for hyper-activation of the heat shock response. An active subset of full-length genes from the primary screen was transfected at multiple concentrations and subjected to two different heat shock temperatures, (A) 43°C and (B) 44°C. The degree of hyper-activation of the response is represented by the intensity of the yellow color. A positive hit is scored if the full-length gene activates the normalized HSP70B reporter by more than two standard deviations above the mean of the negative control (pCMV-Sport6 Empty). The response curve under non-heat shock conditions was monitored but not included in the calculation of the score.

A total of 940 full-length genes that activated the HSP70B reporter by more than 2-fold were selected from the primary screen. The cutoff corresponded to more than 6 standard deviations above the negative expression plasmid control. The selected genes were re-picked from frozen bacterial stocks and the plasmid DNA purified. In the secondary screen, the genes were tested in dose response (3.3, 10, 30, 90 ng per well) while keeping the total amount of DNA constant through the addition of an empty expression plasmid. We performed two independent secondary screens at 43°C and at 44°C for two different heat shock durations (15 and 45 minutes) in duplicate. Following a 6 hour recovery at 37°C, the firefly and *Renilla* luciferase activities were measured. The genes were ranked based on the number of concentrations that hyperactivated the reporter during heat shock. Due to the feedback regulation that exists within the heat shock response, it was hypothesized that overexpression of true regulators would be able to overcome limiting factors within the system and hyperactivate the response while the false positive genes identified in the primary screen would only be able to activate HSF1 under non-stress conditions. Under these conditions, a total of 515 genes hyperactivated the reporter in at least one DNA concentration following heat shock at either 43 or 44°C and at either time point (Fig. 2). The complete results are supplied as supplemental material (Supplemental Table S1).

### Gene Expression Microarray Analysis and RNAi Validation of Potential Heat Shock Regulators

Due to the nature of overexpression screens, only some of the genes identified in the secondary hyper-activation assay are expressed in the cell line under normal growth conditions. In order to validate potential regulators using RNAi, the genes would need to be expressed in the cell line of interest. As a result, gene expression microarray analysis was performed in the HEK-293T cells. The genes that showed significant expression based on standard absent/present calls were cross-referenced with the hits from the secondary hyper-activation screen. From this list, a total of eight genes were selected for RNAi validation (Table 1). Two independent siRNA duplexes were designed for each gene together with two siRNA duplexes for HSF1 as a positive control and two scrambled sequences with identical nucleotide compositions as the HSF1 targets as negative controls. Cells were transfected for 72 hours to allow protein depletion. After 72 hours, the cells were heat shocked and and assayed for firefly and Renilla luciferase 6 hours later. Each well was plate normalized against a scrambled siRNA control. In addition, two constitutively active promoters (thymidine kinase and CMV) driving the same firefly luciferase gene were included on each plate to eliminate any non-specific effects from the luciferase reporter. Luciferase has been previously shown to be a chaperone substrate [26]. A two-sample t-test was performed for each siRNA duplex comparing the difference in HSP70B reporter activity with that from the two constitutively expressed luciferase controls. Based on this comparison, only one of the eight genes, protein kinase C iota (PRKCI), demonstrated a statistically significant decrease in reporter activity (Table 2). The fact that both siRNAs specifically reduce the HSP70B reporter and not the constitutively expressed luciferase controls suggests that PRKCI is required for full activation of HSF1 after heat exposure.

**Table 1:** Rank score of selected genes from the secondary hyper-activation screen and their expression in HEK293T cells using gene expression microarray analysis.

Gene Name	Rank Score in Secondary	U133PLUS 2.0
	Hyper-activation Screen	Probes called (P)resent
	(940 total cDNAs)	
ZCCHC18	27	1^a^
PSMD3	29	1
KCTD10	3	2
KIAA1826	14	2
HMX2	2	1
JMJD2C	7	1
GTSE1	4	5
PRKCI	12	3

a Cross-hybridizes with ZCCHC12.

**Table 2:** Validation of potential HSF1 regulators using RNAi.

	Firefly Luciferase Promoter			Directional FDR Corrected	
	(log_2_Ratio vs. SCRAM_1)^a^			p-value^b^	
siRNA Duplex	HSP70B	TK	CMV	HSP70B vs.TK	HSP70B vs.CMV
PSMD3_1	−1.06±0.20	−1.23±0.53	−0.99±0.61	0.35	−0.4
PSMD3_2	−1.36±0.27	−1.53±0.53	−1.38±0.49	0.35	0.43
ZCCHC12_1	−2.25±0.31	−2.06±0.37	−1.96±0.42	−0.32	−0.25
ZCCHC12_2	−1.59±0.27	−1.43±0.10	−1.37±0.21	−0.24	−0.2
HMX2_1	−1.33±0.31	−1.00±0.32	−0.84±0.53	−0.18	−0.17
HMX2_2	−1.56±0.24	−1.12±0.53	−1.01±0.45	−0.18	−0.099
JMJD2C_1	−0.48±0.23	−0.89±0.34	−0.99±0.24	0.12	0.05
JMJD2C_2	−1.31±0.10	−2.17±0.86	−2.16±0.76	0.12	0.09
PRKCI_1	−0.18±0.05	0.26±0.04	0.42±0.20	**−0.00016**	**−0.0064**
PRKCI_2	−0.77±0.15	−0.12±0.35	−0.21±0.30	**−0.038**	**−0.039**
KCTD10_1	−0.52±0.23	−0.28±0.23	−0.29±0.13	−0.18	−0.14
KCTD10_2	0.39±0.29	−0.05±0.12	0.18±0.22	0.064	0.24
KIAA1826_1	−0.05±0.22	−0.35±0.25	−0.22±0.35	0.14	0.31
KIAA1826_2	−0.07±0.21	−0.13±0.17	0.02±0.29	0.36	−0.36
GTSE1_1	−0.47±0.17	−0.83±0.53	−0.82±0.55	0.21	0.22
GTSE1_2	0.01±0.14	−0.13±0.12	−0.11±0.24	0.17	0.3
SCRAM_2 (neg.)	−0.05±0.18	−0.26±0.17	−0.14±0.23	0.15	0.35
HSF1_1 (pos.)	−2.40±0.22	0.01±0.19	0.11±0.29	**−0.00012**	**−0.00021**
HSF1_2 (pos.)	−3.37±0.25	−0.13±0.32	−0.04±0.24	**−0.00012**	**−0.000078**

a Mean ± SD for 4 biological replicates.

b Statistical significance between the HSP70B reporter and the TK and CMV reporters was calculated using a two sample t-test. The p-values were corrected for multiple comparisons using false discovery rate. Negative p-values denote a reduction in activity versus the control reporter. SCRAM_1 and SCRAM_2 denote negative RNAi controls. HSF1_1 and HSF1_2 denote positive RNAi controls for the reduction of HSF1 activity.

### Quantitative RT-PCR Verification of RNAi Knockdown

**Figure 3:**
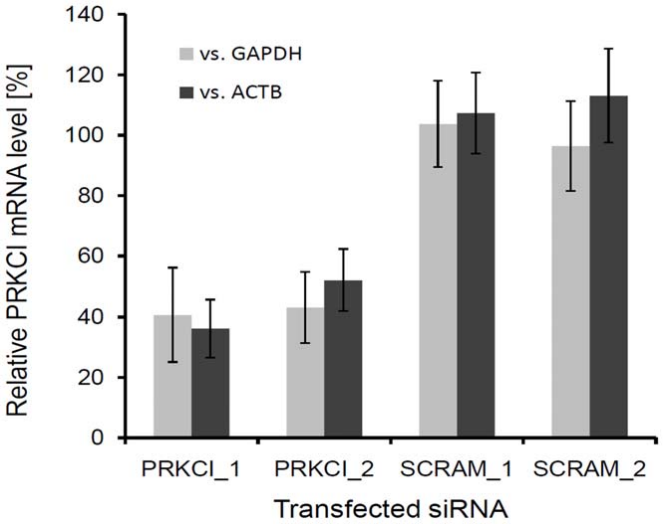
Quantitative RT-PCR verification of PRKCI knockdown by the siRNA duplexes. The bars represent the mean and standard deviations from two independent experiments. PRKCI_1, siRNA duplex #1 targeting PRKCI; PRKCI_2, siRNA duplex #2 targeting PRKCI; SCRAM_1, scrambled siRNA duplex #1; SCRAM_2, scrambled siRNA duplex #2. The light bars represent normalization to GAPDH and the dark bars represent normalization to ACTB.

To verify knockdown of PRKCI mRNA following transfection of the siRNA duplexes, quantitative RT-PCR was performed on the samples and the results normalized to the expression of two different housekeeping genes (Fig. 3). The two siRNA duplexes targeting PRKCI produced an approximate 60% reduction in mRNA levels compared to the scrambled siRNA controls. The reduction was observed regardless of which housekeeping gene was used for normalization.

## Discussion

The identification of potential regulators of the mammalian heat shock response has broader implications than just providing a better understanding of the cellular response to thermal stress. The heat shock response also acts as a surrogate of the general protein quality control system within the cell which plays a significant role in aging and many protein folding diseases as well as the responses to other physical and chemical stressors [16], [27], [28]. In this study, a broad-based functional genomics approach was taken to identify potential regulators of the mammalian heat shock response. The primary screen identified a large number of potential modifiers that were subjected to a secondary screen for hyper-activation of the response under severe heat stress. The secondary screen was used to rank the potential modifiers and gene expression microarray analysis was used to identify which genes were expressed in the experimental cell line. A subset of eight genes were chosen for validation using siRNA knockdown. Of the eight genes, only PRKCI showed a statistically significant reduction in the heat shock response for both siRNA duplexes when compared with controls.

PRKCI is a serine/threonine protein kinase and a member of the atypical protein kinase C sub-group together with protein kinase C eta (PRKCH) and zeta (PRKCZ). PRKCI is expressed across many different tissues and its presence has been shown in several signaling complexes that regulate anchorage-independent growth, resistance to chemotherapeutic apoptosis, cellular motility, cellular polarity, and cellular invasion [29]. The role of PRKCI in these cellular processes has led to its use as a prognostic marker and therapeutic target in non-small cell lung and ovarian cancer [29].

Although previous experiments have shown an involvement of protein kinase C in the regulation of HSF1 [30], [31], no studies other than this one have specifically implicated PRKCI in influencing HSF1 activity. Follow-up co-immunoprecipitation experiments performed in our laboratory did not identify a direct interaction between PRKCI and HSF1 (data not shown). This lack of a direct interaction is supported by previous tandem affinity purification experiments that used PRKCI as bait [32]. Together, these result suggests that PRKCI plays an indirect role in regulating the heat shock response. The data supporting an indirect role come primarily from protein interaction experiments that have demonstrated an interaction between PRKCI and the ubiquitin binding protein sequestosome 1 (SQSTM1) [32], [33], PRKCI and HSP90 [32], and PRKCI and an HSP90 co-factor CDC37 [32]. SQSTM1 is a multifunction adaptor protein that binds polyubiquitin and has been shown to play a significant role in selective autophagic degradation of ubiquitinated targets [34], [35] and the breakdown of misfolded proteins [34]. Since the intracellular concentration of denatured proteins impacts HSF1 activity through sequestration of HSPs [10], [36], [37], an indirect link between PRKCI and HSF1 activity through SQSTM1 is plausible. Finally, PRKCI has been shown to interact with its closely related homologue, PRKCZ [38]. PRKCZ has previously been shown to suppress HSF1 activity [39]. Therefore, through this interaction, PRKCI may reverse the inhibitory effects of PRKCZ on HSF1 activity.

The relatively high hit rate in the primary screen and low validation rate (1/8, 12.5%) point to a number of potential caveats with using an overexpression screen for the heat shock response. First, the heat shock response is part of the cell's protein quality control system and the false positives in the context of the primary screen may be due to nonspecific activation of the heat shock response via overexpressed or misfolded protein. The overexpressed or misfolded protein may act as a chaperone substrate and compete with HSF1 for binding resulting in nonspecific activation of the heat shock response [40]. Second, the use of a native promoter instead of synthetic response elements may have also inflated the number of hits in the screen since a promoter is typically regulated by multiple transcription factors that either bind simultaneously as a complex to a regulatory region or in a sequential manner [41]. This dynamic interplay may be artificially affected in an overexpression screen leading to a high number of hits and lower validation rate. Finally, the use of a mixed full-length gene library containing both human and mouse clones may have contributed to the lower than expected validation rate. Although dramatic species differences haven't been observed in other overexpression screening efforts [20], [22], it is possible that orthologs may play different roles in the heath shock response in each species.

In summary, a series of functional genomic screens were used to identify PRKCI as an regulator of the mammalian heat shock response. Although follow-up studies and literature searches suggest that PRKCI is an indirect regulator of the signaling pathway, additional studies will be required to confirm the precise mechanism of HSF1 regulation. Apart from PRKCI, the functional genomic screens identified other potential regulators that have not yet been validated through loss-of-function and other molecular approaches. Future experiments based on these screens may be used to identify additional therapeutic targets for aging and diseases related to protein folding.

## Materials and Methods

### Full-length Gene Library and Screening Plasmids

Construction and preparation of the full-length cDNA library has been described previously [20]. The final concentration of the library was adjusted to 20 ng/µl. The screening plasmids used in this study were as follows: pHSP70B-luc contains firefly luciferase under the control of 2.3 kb of the highly heat inducible native promoter of the human *HSP70B* gene [25]; pCMV-Sport6-Empty is an empty plasmid vector (Invitrogen, Carlsbad, CA); and pRL-CMV contains *Renilla* luciferase under control of the cytomegalovirus promoter (Promega, Madison, WI).

### Primary HSP70B Reporter Screen

Assay plates containing the purified plasmid DNA were prepared in advance by aliquoting 100 ng of each full-length gene into individual wells of a white 384-well plate (Greiner Bio-One, Monroe, NC). Each plate also included eight wells containing the equivalent amount of the pCMV-Sport6-Empty plasmid for use as a negative control. The DNA containing assay plates were stored at −80°C and equilibrated to room temperature prior to use. A mixture of 10 µl OptiMEM (Invitrogen, Carlsbad, CA) and 0.15 µl LipofectAMINE 2000 (Invitrogen) was added to each well and incubated for 5 min at room temperature. Following incubation, 5 ul OptiMEM containing 20 ng pHSP70B-luc and 5 ng pRL-CMV was added to each well using a Multidrop dispenser (Thermo Scientific, Waltham, MA). The complete transfection mix was incubated for 45 min at room temperature to allow the lipoplexes to form. Following the second incubation, 20 µl DMEM (20% FBS) containing 1×10^5^ HEK293-T cells (ATCC, Manassas, VA) were added to each well using the Multidrop (Thermo Scientific). The assay plates were incubated at 37°C and 5% CO_2_ for 24 hours. Following the incubation, the cell media was removed and 20 µl of cell lysis buffer (25 mM Tris-H_3_PO_4_, pH 7.8; 2 mM 1,2-cyclohexanedinitrilotetraacetic acid; 10% Glycerol; 0.5% Triton X-100; and 2 mM DTT) was added to each well. The plates were incubated for 30 min at room temperature to allow complete cell lysis. Firefly luciferase activity in the cell lysate was assessed following the addition of 20 µl Assay Solution I (20 mM tricine, 0.1 mM EDTA, 1.07 mM (MgCo_3_)_4_ Mg(OH)_2_ • 5H_2_O, 2.67 mM MgSO_4_, 33.3 mM DTT, 270 µM coenzyme A, 470 µM luciferin, 530 µM ATP). *Renilla* luciferase activity was measured following the addition of 20 µl Assay Solution II (1.1 M NaCl, 2.2 mM Na_2_EDTA, 0.22 M KH_2_PO_4_ (pH 5.1), 0.44 mg/ml BSA, 1.3 mM NaN_3_, 1.43 µM coelenterazine). Luminescence was measured using an LJL Analyst plate reader (Molecular Devices, Sunnyvale, CA).

Data extraction, plate normalization and well annotation were carried out using a customized database interface. The firefly to *Renilla* luciferase ratio for each well was log_2_ transformed and plate-normalized to the mean of the plate-matched control wells transfected with the pCMV-Sport6-Empty plasmid. The primary screen was run in duplicate on two separate days. On each day, two technical replicates were run for each plate and the results were averaged. The collection of plate-normalized and log_2_ transformed wells containing pCMV-Sport6-Empty across all plates were utilized to calculate the standard deviation of the mean experimental control for the primary screen. Due to the large number of potential hits, the top 940 hits were selected for further analysis. All 940 hits were greater than six standard deviations away from the mean of the pCMV-Sport6-Empty negative control (Fig. 1A).

### Secondary Gene Dosage and Heat Shock Screens

A sub-library containing 940 plasmids was picked from the complete screening library using a Q-bot (Genetix, Hampshire, UK) and the plasmid DNA re-purified from bacterial cultures using previously described methods [20]. Increasing amounts of each cDNA plasmid (3.3, 10, 30, 90 ng) were added to each well. The total amount of DNA (90 ng) was held constant by the addition of pCMV-Sport6-Empty. On each assay plate, eight wells were filled with only pCMV-Sport6-Empty for use as a negative control and plate normalization. The assay plates were incubated at 37°C and 5% CO_2_ for 24 hours to allow transfection and expression of the transfected genes. Following transfection, the assay plates were wrapped with Parafilm and floated on a revolving water bath. The secondary screen was performed in duplicate at two different temperatures (43°C and 44°C) and with two different heat shock durations (15 and 45 minutes). After heat stress, the plates were returned to 37°C for 6 hours before harvest. The firefly and *Renilla* luciferase activities were measured and normalized to the negative control wells. The rank score for each gene was determined by the number of cDNA concentrations that were able to increase the signal of the HSP70B reporter by more than 2 standard deviations above the experimental mean of the control wells.

### Gene Expression Microarray Analysis

Total RNA was isolated from unstressed HEK-293T using RNeasy columns (Qiagen, Valencia, CA) and the integrity of the RNA was verified spectrophotometrically and with the Agilent 2100 Bioanalyzer (Palo Alto, CA). Double-stranded cDNA was synthesized from 5 µg of total RNA using the One-Cycle cDNA synthesis kit (Affymetrix, Santa Clara, CA). Biotin-labeled cRNA was transcribed from the cDNA using the GeneChip IVT Labeling Kit (Affymetrix). Fifteen µg of labeled cRNA was fragmented and hybridized to Affymetrix Human Genome U133 2.0 arrays for 16 hours at 45°C. The hybridized arrays were washed using the GeneChip Fluidics Station 450 and scanned using a GeneChip 3000 scanner. The gene expression data were normalized using MAS5 and the significance of expression of each gene in the HEK-293T cells was determined using the Affymetrix absent/present calls. The gene expression results have been deposited in the National Center for Biotechnology Information Gene Expression Omnibus and are MIAME compliant (Accession No.: GSE21092).

### RNAi Validation of the Secondary Screening Subset

For each of the eight target genes showing significant expression in HEK-293T cells, two independent siRNA duplexes were tested for their ability to reduce the activity of the HSP70B reporter relative to a scrambled control siRNA (SCRAM). For additional verification, the effect of the siRNA duplexes were evaluated in cells transfected with the firefly luciferase gene expressed from two different constitutive promoters. A total of 0.5 pmoles of each siRNA duplex was added per well and reverse transfected with various reporter vectors as described above. The transfected cells were incubated for 72 hours to allow for protein depletion. Following incubation, the assay plates were placed in a 43°C water bath for 40 minutes and returned to 37°C for 6 hours prior to harvest. Firefly and *Renilla* luciferase activity was measured as described above and normalized using the scrambled siRNA control on each plate. Statistical analysis of each siRNA duplex was carried out using a two sample t-test comparing the HSP70B reporter activity with both the pTK-luc or pCMV-luc plasmids. The corresponding p-values were adjusted for a false discovery rate using the q-value method [42]. Negative q-values denote a reduction of normalized Hsp70 driven firefly luciferase activity relative to either the TK or CMV promoter driven firefly luciferase.

### Quantiative RT-PCR Verification of RNAi Knockdown

HEK-293T cells were reverse transfected with two independent siRNAs targeting PRKCI and 2 scrambled control siRNAs as described previously. The transfected cells were incubated for 72 hours to allow for protein depletion and total RNA was isolated using RNeasy columns (Qiagen). First strand cDNA synthesis was carried out via High Capacity cDNA Reverse Transcription Kit (Applied Biosystems, Foster City, CA). Quantitative RT-PCR was performed using TaqMan Universal PCR Master Mix supplemented with human PRKCI, GAPDH or ACTB specific TaqMan probes (Applied Biosystems). ΔΔCt values of PRKCI specific amplification were calculated versus GAPDH and ACTB probe sets and converted to relative mRNA expression levels.

## Supporting Information

Table S1 Results from the Secondary Screen for Hyper-Activation of the Heat Shock Response Under Stressed Conditions
(0.67 MB XLS)
